# Population pharmacokinetic analysis of phase 1 bemarituzumab data to support phase 2 gastroesophageal adenocarcinoma FIGHT trial

**DOI:** 10.1007/s00280-020-04139-4

**Published:** 2020-09-23

**Authors:** Hong Xiang, Lucy Liu, Yuying Gao, Ago Ahene, Monica Macal, Amy W. Hsu, Lyndah Dreiling, Helen Collins

**Affiliations:** 1 Five Prime Therapeutics, Inc., 111 Oyster Point Blvd, South San Francisco, CA 94080 USA; 2 Shanghai Qiangshi Information Technology Co., Ltd, Shanghai, China; 3 Present Address: TRex Bio, Inc., South San Francisco, CA USA; 4 Present Address: Merck and Co., South San Francisco, CA USA

**Keywords:** Bemarituzumab, Anti-fibroblast growth factor receptor 2b, Population PK analysis, Target-mediated clearance, Dose selection, Gastric and gastroesophageal junction adenocarcinoma

## Abstract

**Purpose:**

To report population pharmacokinetic (PK) analysis of the phase 1 study (FPA144-001, NCT02318329) and to select a clinical dose and schedule that will achieve an empirical target trough concentration (*C*_trough_) for an anti-fibroblast growth factor receptor 2b antibody, bemarituzumab.

**Methods:**

Nonlinear mixed-effect modeling was used to analyse PK data. In vitro binding affinity and receptor occupancy of bemarituzumab were determined. Simulation was conducted to estimate dose and schedule to achieve an empirical target *C*_trough_ in a phase 2 trial (FIGHT, NCT03694522) for patients receiving first-line treatment combined with modified 5-fluourouracil, oxaliplatin and leucovorin (mFOLFOX6) for gastric and gastroesophageal junction adenocarcinoma.

**Results:**

Bemarituzumab PK is best described by a two-compartment model with parallel linear and nonlinear (Michaelis–Menten) elimination from the central compartment. Albumin, gender, and body weight were identified as the covariates on the linear clearance and/or volume of distribution in the central compartment, and no dose adjustment was warranted. An empirical target of bemarituzumab *C*_trough_ of ≥ 60 µg/mL was projected to achieve > 95% receptor occupancy based on in vitro data. Fifteen mg/kg every 2 weeks, with a single dose of 7.5 mg/kg on Cycle 1 Day 8, was projected to achieve the target *C*_trough_ on Day 15 in 98% of patients with 96% maintaining the target at steady state, which was confirmed in the FIGHT trial.

**Conclusion:**

A projected dose and schedule to achieve the target *C*_trough_ was validated in phase 1 of the FIGHT trial which supported selection of the phase 2 dose and schedule for bemarituzumab.

**Electronic supplementary material:**

The online version of this article (10.1007/s00280-020-04139-4) contains supplementary material, which is available to authorized users.

## Introduction

Gastric and gastroesophageal junction adenocarcinoma (GEA) represents the fourth most common cancer worldwide and is a highly lethal disease, with 5-year overall survival (OS) rates below 33% in the United States (US) [1–3]. Chemotherapy as standard first-line treatment has demonstrated an improvement in OS compared to best supportive care and additional progress has been made with the use of targeted therapies, such as trastuzumab and ramucirumab. However, even with these therapies, prognosis is poor. Therefore, the identification of new therapeutics with acceptable toxicities is important for this patient population [4, 5].

The fibroblast growth factor/fibroblast growth factor receptor (FGF/FGFR) pathway can stimulate the transformation and proliferation of tumor cells and angiogenesis. FGF signaling is mediated by a family of transmembrane tyrosine kinase receptors encoded by four distinct genes producing FGF receptor subtypes termed FGFR1–4 [6]. Fibroblast growth factor receptor 2b (FGFR2b), one of 2 FGFR2 splicing variants, is expressed in tissues of epithelial origin (e.g., stomach, skin) with FGF7, FGF10, and FGF22 as three of its major ligands [7]. Alterations in signaling in the FGF/FGFR2 pathway (e.g., overexpression of FGFR2 protein or amplification of *FGFR2* gene) have been associated with GEA, breast, and other cancers and with a decreased prognosis [6, 8–11], suggesting that inhibition of FGFR2 may be a rational target for cancer therapy [12, 13].

Bemarituzumab (FPA144) is a first-in-class humanized Immunoglobulin G1 (IgG1) monoclonal antibody specific to the human FGFR2b receptor that blocks FGF binding to the receptor. It is also glycoengineered for increased affinity for human Fc gamma receptor IIIA (FcγRIIIa) expressed on natural killer cells, resulting in enhanced antibody-dependent cell-mediated cytotoxicity (ADCC) against FGFR2b-overexpressing tumors. These two mechanisms of action of bemarituzumab may together lead to improved OS in patients with GEA whose tumors overexpress FGFR2b.

Bemarituzumab has been evaluated in patients receiving treatment for late-line solid tumors, including those with GEA with or without overexpression of FGFR2b in the FPA144-001 trial (NCT02318329). Promising single agent activity with acceptable tolerability was demonstrated in patients whose GEA overexpressed FGFR2b [14]. GEA tends to be highly heterogeneous within the same tumor, and when present, FGFR2b may not be uniformly distributed throughout the tumor specimen [15, 16]. Combining bemarituzumab with chemotherapeutic agents that will target FGFR2 negative tumor cells is likely to improve the clinical benefit over bemarituzumab alone. Currently, bemarituzumab is being evaluated in combination with modified 5-fluourouracil, oxaliplatin, and leucovorin (mFOLFOX6) in the phase 2 placebo-controlled trial, A Study of Bemarituzumab Combined with Modified FOLFOX6 in Gastric/Gastroesophageal Junction Cancer (FIGHT, NCT03694522).

Dose selection is frequently a challenge for novel biological oncology drugs because the maximum tolerated dose approach may identify a dose that is significantly higher than the dose required to achieve maximum efficacy [17]. There are additional challenges for an orphan drug like bemarituzumab because of the lower prevalence of tumors expressing the target FGFR2b, which may limit the feasibility of an extensive clinical dose-ranging study. Therefore, an efficient approach was needed to advance bemarituzumab dose selection. To achieve this goal, multiple factors had to be considered, including population pharmacokinetic analysis (PopPK), which is the focus of this manuscript. On the basis of the mechanism of action for bemarituzumab (including blocking of FGFR2b binding to its ligands), it is projected that keeping trough concentration (*C*_trough_) above a threshold to saturate the target between-dose interval might provide maximum anti-tumor activity. Therefore, an empirical target *C*_trough_ estimated from in vitro data was included as an exposure target to support dose selection. This approach was used in multiple targeted antibody therapies including bevacizumab and trastuzumab as well as immunotherapies, such as ipilimumab [18–20]. In addition, a concentration associated with a high percentage of saturation of target-mediated clearance for bemarituzumab can be used as the target *C*_trough_.

Here, we describe results from the PopPK analysis of bemarituzumab using serum concentration data from a first-in-human phase 1 study (FPA144-001) to support dose selection for the phase 2 trial. The key objectives of the analysis were to assess pharmacokinetic (PK) and covariates of bemarituzumab in humans using PopPK analysis, to describe the in vitro data used to derive an empirical target *C*_trough_, to report the simulation results which identified the dose and regimen necessary to achieve an empirical target *C*_trough_ in the majority of patients, and to provide clinical data to support the projection from the simulation.

## Materials and methods

### Antibody reagents

Bemarituzumab was produced in a Chinese hamster ovary cell line that lacks the *FUT8* gene (α1, 6-fucosyltransferase) at Five Prime Therapeutics, Inc. (South San Francisco, CA) for nonclinical studies, at AbbVie Bioresearch Center (Worcester, MA) for the FPA144-001 trial, and at Patheon (St. Louis, MO) for the FIGHT trial.

### Bemarituzumab serum concentration in humans

Human serum concentration data were collected from 75 patients enrolled in the FPA144-001 clinical trial; 22 patients with a variety of solid tumors and 53 patients with GEA. Among the 75 patients, 27 were enrolled in the dose escalation portion of the trial and received a 30-min intravenous (IV) infusion of bemarituzumab once every 2 weeks (Q2W) in a range of doses [0.3 (*n* = 3), 1 (*n* = 4), 3 (*n* = 4), 6 (*n* = 4), 10 (*n* = 9), and 15 (*n* = 3) mg/kg; Part 1]. An additional 48 patients received 15 mg/kg Q2W in a 30-min IV infusion (Part 2). Each cycle had two doses with a Q2W dosing regimen. Three to five serum concentration samples were collected within 8 h on the day of dosing followed by collections on Days 2, 4, and 8 post-first dose of Cycle 1. Additional collections were done both before and at the end of infusion for the second dose of Cycle 1 and the first dose of Cycles 2 through 5, every other cycle thereafter, and at the End of Treatment Follow-up Visit. Part 2 had the same serum concentration sample collection time points without collection of samples on Days 2 and 4 post-first dose.

FIGHT is a phase 2 trial preceded by dose-finding in phase 1. *C*_trough_ data collected during Cycle 1 through Cycle 5 from ten patients (*n* = 3 for 6 mg/kg and *n* = 7 for 15 mg/kg Q2W with a single dose of 7.5 mg/kg on Cycle 1 Day 8) in phase 1 of the FIGHT trial made up the observed serum concentration data displayed in Fig. 4.

### Anti-drug antibody in humans

Anti-drug antibody (ADA) samples were collected from all patients in study FPA144-001 prior to dosing on Day 1 of Cycles 1 to 5, every other cycle after Cycle 5, and at the End of Treatment Follow-up Visit.

### Determination of serum concentration of bemarituzumab in humans

The bemarituzumab serum concentration in humans was quantitatively measured at ICON Laboratory Services, Inc. (Whitesboro, NY) with a validated ELISA in which bemarituzumab was captured by plate-bound FGFR2b-Fc and detected by an anti-idiotypic antibody coupled to HRP. The LLOQ of the assay was 0.125 µg/mL. The accuracy expressed as percent relative error ranged from 0.250% to 4.80%. The intra- and inter-assay ranges were − 0.240% to − 4.80% and 5.74% to 7.45%, respectively.

### Measurement of anti-drug antibody in patients

A validated bridging electrochemiluminescence assay that utilizes Meso Scale Discovery (MSD) technology was used to detect ADA in patient serum samples. The samples were acidified, then neutralized in a reaction mixture containing ruthenylated bemarituzumab and biotinylated bemarituzumab. After a 75-min incubation, the samples were transferred onto a streptavidin coated MSD assay plate. The plate was washed and a tripropylamine (TPA)-containing MSD read buffer added. In the presence of TPA, ruthenium produces a chemiluminescent signal when voltage is applied. Only samples that contained antibody bound to both biotinylated bemarituzumab and ruthenylated bemarituzumab produced the chemiluminescent signal, which was proportional to the amount of anti-bemarituzumab antibody present. The assay sensitivity was 32.7 ng/mL relative to the rabbit anti-FPA144 positive control. Drug tolerance was 2 µg/mL in the presence of 800 ng/mL of rabbit anti-bemarituzumab antibody.

### Human population pharmacokinetic analysis

A PopPK model was developed to describe serum bemarituzumab concentration–time profiles. Various PK structural models were tested, including a one-compartment, two-compartment, two-compartment with time-varying clearance, and two-compartment with linear and nonlinear elimination components. The PK of bemarituzumab in the tested clinical dose range was best described by a two-compartment model with parallel linear and nonlinear (Michaelis–Menten) elimination pathways from the central compartment using the differential equations below [21, 22]:

1 $$\frac{{{\text{d}}A_{{\text{c}}} }}{{{\text{d}}t}} = - \left[ {\left( {\frac{{V_{{\max}} }}{{K_{{\text{m}}} + \frac{{A_{{\text{c}}} }}{{{\text{Vc}}}}}}} \right)/V_{{\text{c}}} + \frac{{{\text{CL}}}}{{V_{{\text{c}}} }} + \frac{Q}{{V_{{\text{c}}} }}} \right]{ } \times { }A_{{\text{c}}} + \frac{Q}{{V_{{\text{p}}} }} \times { }A_{{\text{p}}}$$

2 $$\frac{{{\text{d}}A_{{\text{p}}} }}{{{\text{d}}t}} = \frac{Q}{{V_{{\text{c}}} }} \times { }A_{{\text{c}}} - \frac{Q}{{V_{{\text{p}}} }} \times { }A_{{\text{p}}}$$

where *A*_c_ and *A*_p_ are the amount of drug in central and peripheral compartments, respectively. *V*_max_ represents the maximum drug elimination by nonlinear clearance, and *K*_m,_ Michaelis–Menten constant, indicates the drug concentration at 50% *V*_max_. CL and *Q* represent linear clearance and distribution clearance, respectively, while *V*_c_ and *V*_p_ represent volume of distribution in central and peripheral compartments, respectively.

After an optimal structural model was identified (final base model), the effect of covariates (Table 1) including baseline demographic information, renal function, hepatic function, disease status, baseline tumor measurements, Eastern Cooperative Oncology Group (ECOG) status, and FGFR2b expression in patients with GEA were evaluated for their impacts on the CL and V_c_. FGFR2b high was defined as ≥ 10% of tumor cells with 3 + membranous staining using a centrally performed validated laboratory-developed prototype immunohistochemistry assay (LabCorp, Burlington, NC). Correlations between the PK parameters and the covariates were explored graphically, followed by linear regression (continuous covariates) and analysis of variance (ANOVA) testing (categorical covariates) using R software. These analyses were conducted on individual random effects (ETAs) for CL and *V*_c_. Only covariates that showed a significant (*p* < 0.01) effect on the random effect, and that could be meaningfully explained from both a clinical and scientific perspective, were examined further using NONMEM. Covariates were selected based on their potential clinical relevance. The possible physiological basis for commonly evaluated covariates such as body size, age, gender, race, ADA, serum albumin, creatinine clearance, etc., were summarized by Thomas and Balthasar [23]. Selection of the final covariate model (final PopPK model) was determined for its significance on the basis of likelihood ratio test at the *p* < 0.01 for forward inclusion and *p* < 0.001 for backward deletion.

**Table 1:** Covariate values of bemarituzumab population pharmacokinetic dataset

Variable	Study FPA144-001 (*n* = 75)
Age (year), median (range)	58 (25, 86)
Weight (kg), median (range)	61.4 (35.5, 148)
Gender, *n* (%)	
Female	42 (56%)
Male	33 (44%)
Race, *n* (%)	
White	29 (38.7%)
American Indian or Alaska Native	1 (1.33%)
Asian	44 (58.7%)
Black or African American	1 (1.33%)
Albumin (g/dL), median (range)	3.7 (1.9, 4.6)
Creatinine Clearance (mL/min), median (range)	75.3 (26.4, 200)
Total Bilirubin (mg/dL), median (range)	0.4 (0.1, 2.0)
ALT (U/L), median (range)	19 (6, 74)
AST (U/L), median (range)	24 (7, 106)
Baseline Tumor Size (mm), median (range)	50 (10, 244)
Tumor type, *n* (%)	
Gastric cancer	53 (70.7%)
Other solid tumors	22 (29.3%)
ECOG status, *n* (%)	
0	23 (30.7%)
1	52 (69.3%)
FGFR2b Expression in patients with gastric and gastroesophageal junction adenocarcinoma, *n* (%)	
FGFR2b high	26 (49%)
FGFR2b other	27 (51%)

The final PopPK model was evaluated with multiple internal model validations, including goodness-of-fit diagnostics, prediction-corrected visual predictive check (pcVPC) plots, numerical predictive check, bootstrap, and shrinkage assessments. The pcVPC was created to assess the predictive ability of the model. A total of 1000 replicates of the trials were simulated using the individual dosing history and covariates, the typical parameter estimates, and random sampled interindividual variability and residual errors. The 2.5th, 50th, and 97.5th percentiles of the observed data were overlaid on the 90% confidence interval (CI) of the 2.5th, 50th, and 97.5th simulated percentiles, and a visual inspection was performed. The sensitivity analysis was performed for the final PopPK model to examine the contribution of significant baseline covariates to the overall variability of the steady-state exposures including area under concentration–time curve at steady state (AUC_ss_), maximum serum concentration at steady state (*C*_max ss_), and trough concentration at steady state (*C*_trough ss_) after Q2W dosing of 15 mg/kg bemarituzumab.

The PopPK analysis was performed using the nonlinear mixed effects modeling approach with the first-order conditional estimation with interaction method. Model parameter estimation and evaluation were implemented with NONMEM 7 (v. 7.3.0; ICON Development Solutions, Ellicott City, MD) with an Intel Fortran Compiler (v. 10.1.021; Intel, Santa Clara, CA), Perl-speaks-NONMEM (PsN, v. 3.2.12; Uppsala University, Uppsala, Sweden), and R 3.3.1.

### Binding affinity using surface plasmon resonance

The binding affinity of bemarituzumab for human FGFR2b ECD-IgG1 Fc fusion protein (FGFR2b-Fc) was measured using surface plasmon resonance (SPR, Biacore T100, GE Healthcare Life Science, Marlborough, MA). Bemarituzumab was immobilized on a dextran chip using an amine coupling kit and 100 mM ethylenediamine in 100 mM Sodium Borate, pH 8.0, was used as the blocking reagent. Six different concentrations (0 nM to 500 nM) of FGFR2b-Fc proteins were diluted in HEPES buffered saline with 0.05% surfactant P20 running buffer and flowed over the immobilized antibody.

To determine binding affinity for FcγRIIIa (V158) by SPR, bemarituzumab was captured on the chip via protein A. Protein A was covalently attached to a dextran chip using the same protocol as above. Five concentrations (0 nM to 1000 nM) of FcγRIIIa (V158) were diluted in running buffer and flowed over the captured antibody.

The association constant, dissociation constant, and affinity for bemarituzumab binding to human FGFR2b and human FcγRIIIa (V158) were calculated using the Biacore T100 Evaluation Software 1:1 binding model.

### Cell lines

OCUM-2M is a *FGFR2* gene-amplified, FGFR2b protein overexpressing gastric cancer cell line obtained from Public University Corporation Osaka City University, Japan (Source: Dr Masakazu Yashiro). HSC-39 is a *FGFR2*-amplified gastric cell line and was kindly provided by Rebecca Fitzgerald (Hutchison/MRC Research Centre, Cambridge, UK). Gastric cell lines SNU-16 (with *FGFR2* amplification) and NCI-N87 (without *FGFR2* amplification or overexpression) were commercially acquired (ATCC, Manassas, VA). All four cell lines were mycoplasma negative tested by IDEXX Laboratories (Columbia, MO) using a real-time polymerase chain reaction.

### Gastric cell lines-based binding

The cell surface expression of FGFR2b on OCUM-2 M, HSC-39, SNU-16, and NCI-N87 cells was calculated after incubation with 50 µg/mL of either bemarituzumab or human IgG1 (human anti-hen egg lysozyme, Five Prime Therapeutics, Inc). Unbound antibody was washed with 1 × phosphate-buffered saline with 2% fetal bovine serum (VWR Visalia, CA) and the cells were incubated with mouse anti-human IgG1-PE (Clone HP6001, lot L3117-M839, SouthernBiotech, Birmingham, AL). Cells were washed and data acquired on the BD LSRII Flow Cytometer (BD, San Jose, CA). Quantum Simply Cellular anti-mouse IgG Bang Beads (815B lot 12,380, Bangs Laboratories, Fishers, IN) were stained in parallel according to the manufacturer’s instructions and acquired on the BD LSRII Flow Cytometer. The antibody binding capacity (ABC) was calculated using the Bang Laboratories QuickCal Template.

Separately, 2 × 10^5^ OCUM-2 M, HSC-39, SNU-16, or NCI-N87 cells were incubated with varying concentrations of bemarituzumab or human IgG1, washed, stained with a mouse anti-human IgG1 PE secondary antibody (clone HP6001; SouthernBiothech, Birmingham, AL), washed, and acquired on the BD LSRII Flow Cytometer. A 4-parameter logistical regression curve fit for acquired mean fluorescence intensity versus bemarituzumab concentration data in semi-log plot was applied in GraphPad PRISM (La Jolla, CA) to estimate receptor occupancy.

### Human pharmacokinetic simulation

Bemarituzumab exposures were simulated using the final PopPK model with different dose regimens. For each dose regimen, 1000 simulated model parameters constructed the distribution of model predictions for a typical population (61 kg male patients with albumin of 3.7 g/dL). The area under the concentration–time curve (AUC), *C*_max_, and *C*_trough_ after single and multiple treatment for each of the dose regimens were computed. The simulation was done for the dose cohorts tested in the safety lead-in phase 1 portion of the FIGHT trial: 6 mg/kg Q2W and 15 mg/kg Q2W with a single dose of 7.5 mg/kg on Cycle 1 Day 8.

## Results

### Bemarituzumab serum concentration in humans

Bemarituzumab serum concentration versus time data (group mean ± SD) from Cycle 1 dose 1 in the FPA144-001 trial displayed a typical antibody serum concentration profile with a short distribution phase followed by a long elimination phase [14]. Bemarituzumab demonstrated dose dependent clearance in patients with solid tumors including GEA, with faster clearance at lower doses, suggesting target-mediated drug disposition. Clearance appeared nonlinear from 0.3 mg/kg to 1 mg/kg and approximately linear from 1 mg/kg to 15 mg/kg.

### The anti-drug antibody impact on bemarituzumab pharmacokinetics

A total of 75 patients from the FPA144-001 trial were tested for ADA. The patients were exposed to bemarituzumab for up to 966 days (median = 55 days). No patient sample was confirmed to be ADA positive after administration of bemarituzumab. A predose sample before bemarituzumab administration from one patient was ADA positive; however, all tested ADA samples post-bemarituzumab administration for this patient were negative. The predose ADA positive signal did not have a visible impact on the PK profile of this patient in comparison with the PK profiles from the other patients treated at the same dose level.

### Population pharmacokinetic analysis

The development dataset for the final model included 814 bemarituzumab serum concentration data from 75 participants in the phase 1 study FPA144-001. A two-compartment model with parallel linear and nonlinear (Michaelis–Menten) elimination from the central compartment best described the bemarituzumab serum concentration data. No time-varying CL was identified after bemarituzumab administration. Covariate analysis was conducted to understand the impact of the covariate values (Table 1) on the CL and *V*_c_ of bemarituzumab. The forward addition and backward deletion based on the final base model identified body weight, albumin, and gender as statistically significant covariates for bemarituzumab disposition parameters of CL and *V*_c_, and this model is referred to as the final PopPK model:

3 $${CL}_{i}=exp\left({\theta }_{1}+{\theta }_{7}\cdot \left(\frac{\mathrm{Weight}}{61}\right)+{\theta }_{9}\cdot (\frac{\mathit{ALB}}{3.7})+{}{\eta_{CL}}\right)$$

4 $${{V}_{C}}_{i}=exp\left({\theta }_{2}+{\theta }_{8}\cdot \left(\frac{\mathrm{Weight}}{61}\right)+{\theta }_{10}\cdot \mathrm{female}+{}{\eta_{Vc}}\right)$$

where CL_i_ is the individual linear clearance; *V*_ci_ is the individual volume of distribution in the central compartment, *η*_CL_ and *η*_Vc_ are interindividual variability of CL and *V*_c_, respectively.

The final PopPK model estimated a typical CL, saturation of target-mediated drug disposition, of 0.331 L/day, *V*_c_ of 3.70 L, *Q* of 0.788 L/day, *V*_p_ of 2.05 L, *V*_max_ of 1.70 µg/day, and *K*_m_ of 4.58 µg/mL. The estimated linear clearance half-life was 12.8 days. The interindividual variability on CL, *V*_c_, and *V*_p_ were 27.1%, 17.3%, and 60.0%, respectively.

**Fig. 1:**
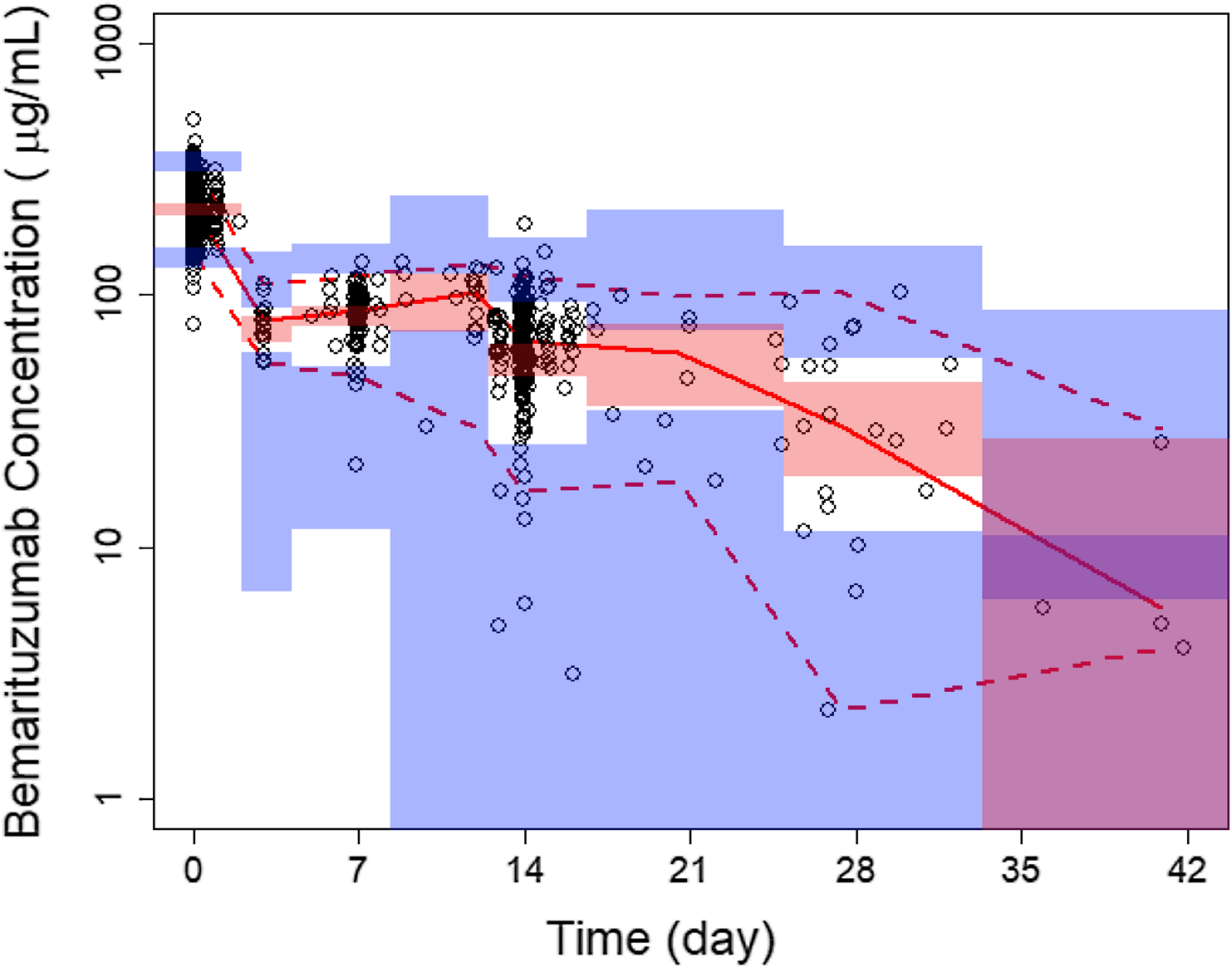
Prediction-corrected VPC of bemarituzumab serum concentration–time profile across dose groups. Black open circles are observed serum concentrations, solid red line represents the median observed value, and dashed red lines represent 2.5th and 97.5th percentile of the observed values, respectively. Pink shaded areas represent the spread of the median predicted values (5th to 95th percentile), and blue shaded areas represent the spread (5th and 95th percentile) of the 2.5th and 97.5th predicted percentile concentrations

Bootstrapping of 1000 datasets resulted in median parameter estimates and 95% CI similar to the estimates from the original dataset (Table 2), indicating that the final PopPK model provided good precision for parameter estimation. Goodness-of-fit plots showed agreement between predicted and observed concentrations of bemarituzumab with no apparent bias in residual plots over time or across population-predicted concentrations (Supplementary Fig. 1). pcVPC plots (Fig. 1) demonstrated that the final PopPK model could reasonably describe the central tendency and variability of the bemarituzumab pharmacokinetic data.

**Table 2:** Summary of population pharmacokinetic parameters

Parameter description	Base model estimates (%RSE)	Final model estimates (%RSE)	Bootstrap estimate median (2.5–97.5%tiles)
*V*_max_ (μg/day)	2.75 (21.6%)	1.70 (14.3%)	1.82 (0.0901; 7.55)
*K*_M_ (μg/mL)	4.64 (42.5%)	4.58 (15.1%)	5.24 (0.533; 24.7)
Linear clearance, CL (L/day)	0.331 (4.73%)	0.331 (3.55%)	0.327 (0.275; 0.364)
Influence of body weight on CL	–	0.601 (19.5%)	0.641 (0.355; 0.991)
Influence of albumin on CL	–	− 0.776 (19.9%)	− 0.777 (− 1.31; − 0.262)
Volume of central compartment, *V*_c_ (L)	3.43 (2.76%)	3.70 (2.98%)	3.70 (3.51; 3.92)
Influence of body weight on *V*_c_	–	0.303 (24.8%)	0.306 (0.109; 0.471)
Influence of sex on *V*_c_	–	− 0.191 (23.4%)	− 0.194 (− 0.284; − 0.114)
Distribution clearance, *Q* (L/day)	0.772 (10.6%)	0.788 (8.99%)	0.791 (0.599; 1.11)
Volume of peripheral compartment, *V*_p_ (L)	2.07 (8.25%)	2.05 (7.73%)	2.08 (1.76; 2.42)
Interindividual variability of *V*_max_	113 (32.6%)	128 (23.3%)	122 (65.9; 437)
Interindividual variability of CL	36.0 (20.1%)	27.1 (19.7%)	25.5 (18.4; 31.7)
Interindividual variability of *V*_c_	22.2 (18.2%)	17.3 (19.1%)	16.8 (13.7; 19.9)
Interindividual variability of *V*_p_	59.3 (25.1%)	60.0 (24.1%)	56.0 (38.3; 74.9)
Covariance between CL and *V*_c_	0.0319 (36.2%)	0.0141 (46.1%)	0.0137 (0.00111; 0.0254)
Residual variability (%CV)	14.4 (5.61%)	14.5 (5.89%)	14.4 (13.0; 15.7)

$$CL_{i} \left( {{\text{L}}/{\text{hr}}} \right) = \exp \left( { - 4.284 + 0.601 \times \log \left( {\frac{{{\text{WT}}}}{61}} \right) - 0.776 \times \log \left( {\frac{{{\text{ALB}}}}{3.7}} \right) + \eta_{CL,i} } \right)$$

$$Vc_{i} \left( L \right) = \exp \left( {1.308 + 0.303 \times \log \left( {\frac{\text{WT}}{61}} \right) - 0.191 \times {\text{Female}} + \eta_{Vc,i} } \right)$$

**Fig. 2:**
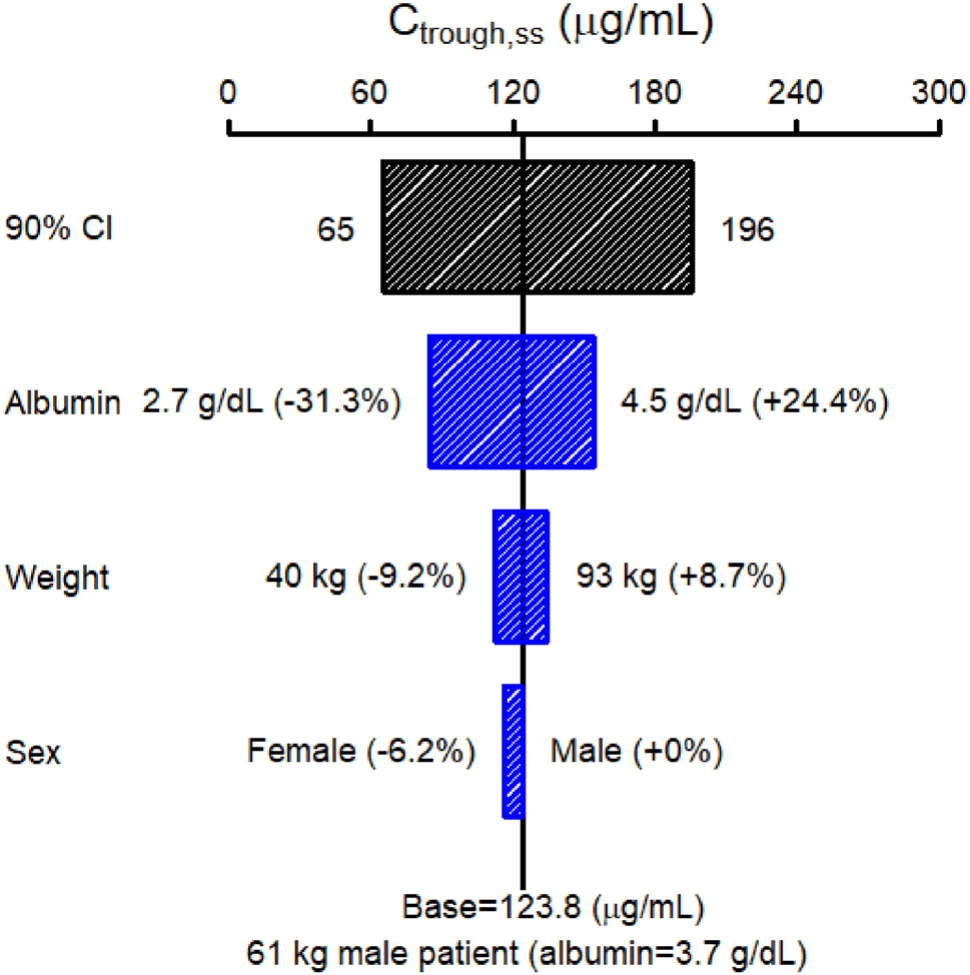
Sensitivity plot comparing the effect of covariates on *C*_trough ss_ of bemarituzumab. Base, as represented by the black vertical line and value, refers to the predicted typical *C*_trough ss_ of bemarituzumab in a 61 kg male patient with an albumin of 3.7 g/dL kg after continuous Q2W dosing of 15 mg/kg bemarituzumab for 6 months. The black horizontal bar with values at each end shows the 5th to 95th percentile *C*_trough ss_ range across the entire population. Each blue bar represents the influence of a single covariate on the *C*_trough ss_. The label at the left end of the bar represents the covariate being evaluated. The upper and lower values for each covariate capture 90% of the plausible range in the population. The length of each bar describes the potential impact of that particular covariate on bemarituzumab *C*_trough ss_ with the percentage value in the parentheses at each end representing the percent change of *C*_trough ss_ from the base. The most influential covariate is at the top of the tornado plot

Body weight was identified as a covariate for both CL and *V*_c_, while albumin was a covariate for CL and gender was a covariate for *V*_c_. Patients with higher body weight had statistically larger *V*_c_ and faster CL. Lower albumin had statistically faster CL and male patients had a higher *V*_c_. Due to the limited number of patients (*n* = 75), the covariate relationship should be confirmed in future studies. The sensitivity analysis suggested that albumin is the most important factor influencing the *C*_trough ss_ of bemarituzumab. The population-predicted 5th and 95th percentile of total *C*_trough ss_ for the actual patients’ Q2W dosing of the 15 mg/kg were 65 and 196 µg/mL, respectively, corresponding to − 47.5% and 58.3% variation around the *C*_trough ss_ predicted for a typical patient (123.8 µg/mL). Of note, the extreme albumin (5th and 95th percentiles) corresponded to as high as − 31.3% and 24.4% variation for *C*_trough ss_ (Fig. 2). Body weight had a mild impact based on the sensitivity analysis (− 9.2% and 8.7% variation on *C*_trough ss_; Fig. 2). Gender was also an influential factor for the *C*_trough ss_ with a − 6.2% variation for female compared to male patients (Fig. 2). Albumin was the most influential factor for AUC_ss_ with − 21.7% and 16.4% variation while body weight had the greatest impact on *C*_max ss_ with − 20% to 25.7% variation (data not shown).

### An empirical target trough concentration based on in vitro data

The affinity of bemarituzumab for FGFR2b and FcγRIIIa (V158) was measured by SPR. The binding affinity of bemarituzumab for FGFR2b was 0.58 nM and for FcγRIIIa (V158) was 9.2 nM. Therefore, 95% and 99% receptor occupancy by bemarituzumab for FGFR2b is estimated to be achieved at concentrations ≥ 1.59 µg/mL and ≥ 8.26 µg/mL, respectively, and 95% and 99% receptor occupancy for FcγRIIIa (V158) is estimated to be achieved at concentrations of 25.1 µg/mL and 131 µg/mL, respectively, using the following equation:

1 $$\% {\rm Receptor\,occupancy}=(C/{\rm KD}+C) \times100.$$

where *C* is the concentration of the molecule and KD is the affinity of the molecule for its ligand/binding partner.

**Fig. 3:**
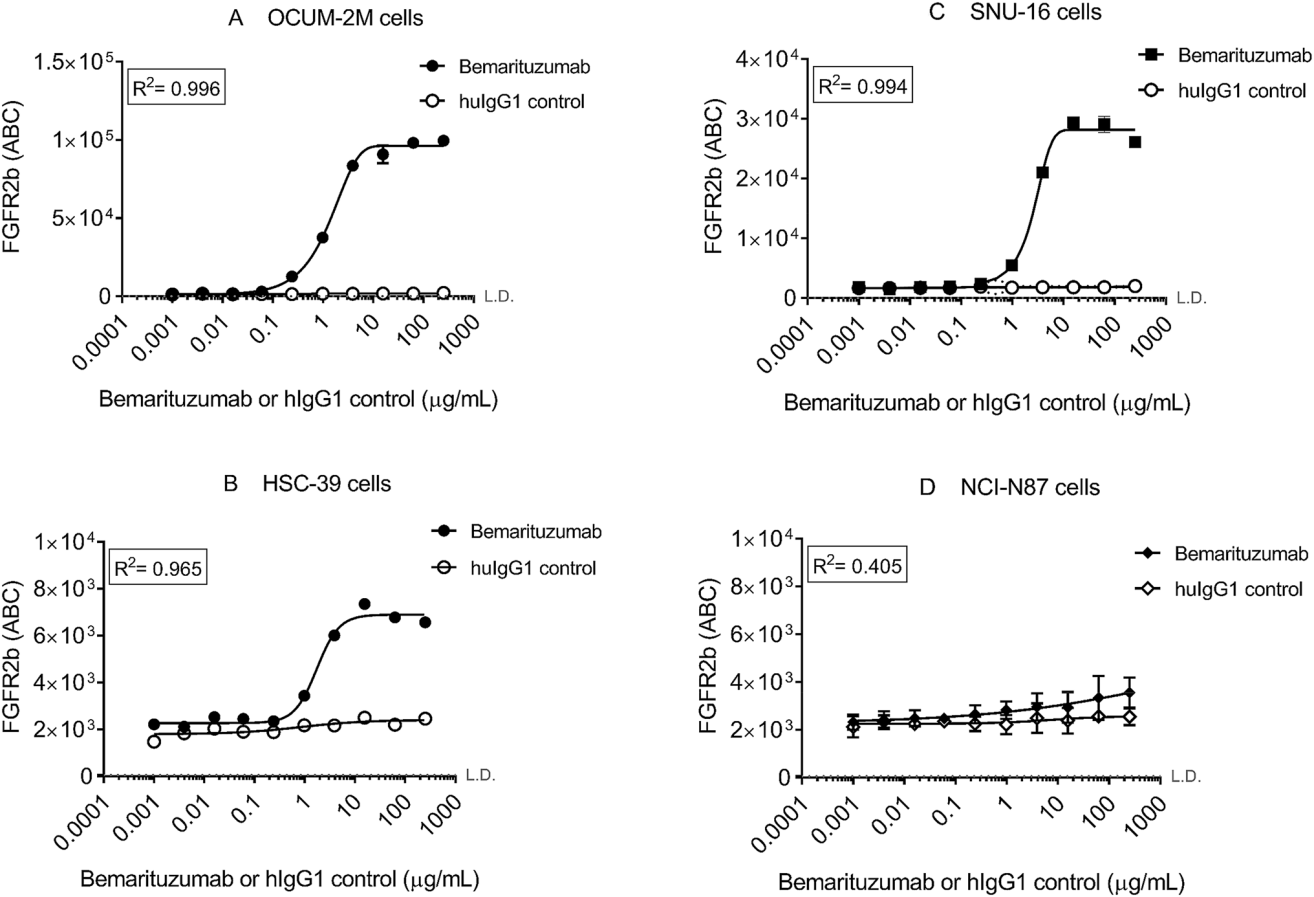
In vitro cell-based binding for bemarituzumab using gastric cell lines. Mean fluorescence intensity (ABC) versus bemarituzumab concentration profile from receptor occupancy study in vitro are presented for FGFR2b-amplified cell lines OCUM-2 M, HSC-39, and SNU-16 in Figs. 3A, 3B, and 3C, respectively. NCI-N87 is presented in 3D as a negative control. Symbols represent observed data (*n* = 3 per time point). *L.D.* limit of detection, which is at 62 for ABC

Among 3 FGFR2-amplified cell lines tested, FGFR2 expression level on the cell surface demonstrated the following order: OCUM-2 M, SNU-16, and HSC-39. The mean EC_50_ is similar among these three cell lines with values at 1.37 ± 0.0525 µg/mL, 1.81 ± 0.0289 µg/mL, and 2.53 ± 0.170 µg/mL for OCUM-2 M, HSC-39, and SNU-16, respectively (Fig. 3). The EC_95_ is 26.1 ± 1.00 µg/mL, 34.4 ± 0.548 µg/mL, and 48.1 ± 3.24 µg/mL, respectively.

Therefore, an empirical targeted *C*_trough_ of 60 µg/mL was selected, which would achieve > 95% receptor occupancy based on binding affinity and in vitro cell-based binding data from multiple FGFR2-amplified cell lines.

### Simulation to support dose and regimen selection for bemarituzumab in combination with modified FOLFOX6 in the FIGHT trial

**Fig. 4:**
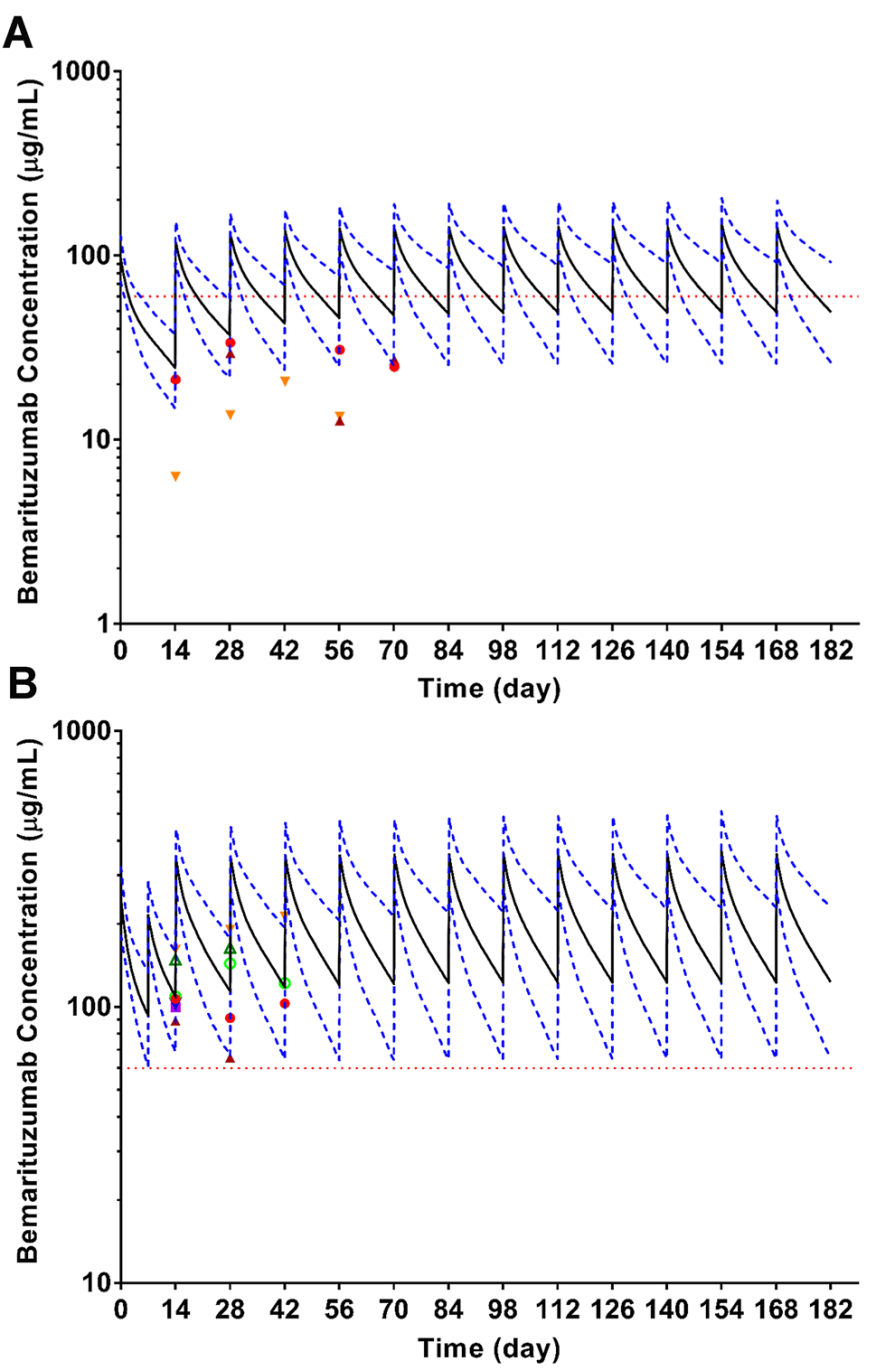
Simulation results using PK parameters obtained from PopPK analysis versus observed *C*_trough_ from phase 1 of the FIGHT trial. Simulated serum concentration versus time profiles of bemarituzumab after continuous Q2W dosing of bemarituzumab at 6 mg/kg (**a**) or 15 mg/kg Q2W with a single dose of 7.5 mg/kg on Cycle 1 Day 8 (**b**) for 6 months based on the final PopPK model. For each dose regimen, 1000 simulated model parameters constructed the distribution of model predictions for a typical population (61 kg male patients with albumin of 3.7 g/dL). In each figure, solid black line represents the median predicted value and dashed blue lines represent the 5th and 95th percentile of the predicted concentrations and dashed red line is for *C*_trough_ of 60 µg/mL. Each symbol represents the individual bemarituzumab C_trough_ (µg/mL) in the presence of mFOLFOX6 at 6 mg/kg (**a**, *n* = 3) and 15 mg/kg Q2W with a single dose of 7.5 mg/kg on Cycle 1 Day 8 (**b**, *n* = 7), respectively

Using the final PopPK model, simulations were performed to determine whether alternative dosing schedules would allow patients to more rapidly reach target *C*_trough_ of ≥ 60 µg/mL for a typical population (61 kg male patients with albumin of 3.7 g/dL). Accordingly, the dose cohorts tested in the safety lead-in phase 1 portion of the FIGHT trial were 6 mg/kg Q2W and 15 mg/kg Q2W with a single dose of 7.5 mg/kg on Cycle 1 Day 8. If the last dose cohort was not tolerable, then the Cycle 1 Day 8 dose was to be omitted and 15 mg/kg Q2W would be tested as a de-escalation dose cohort. The serum concentration versus time profiles for the two dose cohorts in the phase 1 portion of the FIGHT trial were projected (Fig. 4). The addition of a single dose of 7.5 mg/kg on Cycle 1 Day 8 for 15 mg/kg Q2W was predicated to allow a majority of patients (98%) to achieve target *C*_trough_ by Day 15, compared to 96% achieving target *C*_trough_ by week 10 with good tolerance, thereby shortening the time to reach the target *C*_trough_.

### PK profile of bemarituzumab is not affected by addition of mFOLFOX6

Bemarituzumab in combination with mFOLFOX6 had an acceptable toxicity profile in previously treated patients with GEA in the phase 1 safety lead-in portion of the FIGHT trial at its highest dose tested-15 mg/kg Q2W with a single dose of 7.5 mg/kg on Cycle 1 Day 8 [24]. Bemarituzumab concentration was not affected by the presence of mFOLFOX6. In addition, *C*_trough_ concentration from all seven patients who received 15 mg/kg with a single dose of 7.5 mg/kg on Cycle 1 Day 8 achieved target *C*_trough_ of 60 µg/mL (Fig. 4b).

## Discussion

Bemarituzumab, an FGFR2b inhibitor, demonstrated promising monotherapy activity in late-line GEA [14]. A phase 2 trial (FIGHT) was designed to evaluate bemarituzumab combined with mFOLFOX6 chemotherapy in patients with previously untreated GEA whose tumors overexpress FGFR2b or have *FGFR2* amplification, however, efficient dose selection was required. A PK simulation in combination with an empirical target *C*_trough_ obtained from in vitro data was used to support the selection of the maximum test dose at 15 mg/kg Q2W with a single dose of 7.5 mg/kg on Cycle 1 Day 8 with mFOLFOX6 in the phase 1 safety lead-in portion of the FIGHT trial. This dose and schedule were selected for the phase 2 evaluation following the observed PK and safety data from phase 1 portion of FIGHT trial [24].

This is the first report of bemarituzumab PopPK analysis and covariate assessment of the phase 1 data. Bemarituzumab PK was best described by a two-compartment model with parallel linear and nonlinear (Michaelis–Menten) elimination from the central compartment. Target-mediated clearance observed with bemarituzumab has been reported for multiple other antibodies, such as cetuximab, panitumumab, and anti-NRP1 [25–27].

Covariates impacting CL and *V*_c_ are variable for targeted antibodies. For example, body weight and gender were identified as the most significant covariates for CL and *V*_c_ of bevacizumab while baseline serum albumin and lean body weight were identified as significant covariates for pertuzumab CL [28, 29]. None of these covariates for bevacizumab or pertuzumab are significant enough to require a dose adjustment in the clinic for a specific patient population. The covariate analyses for bemarituzumab similarly did not identify any covariates for exposure which would require a dose adjustment. Among 13 covariates evaluated (Table 1), only 3 had a significant impact on either CL or *V*_c_. Body weight impacted both CL and *V*_c_, albumin impacted CL, and gender impacted *V*_c_. Sensitivity analyses indicated that the effects of these covariates on steady-state exposures including *C*_trough ss_, AUC_ss_, and *C*_max ss_ were small compared to the overall between-subject variability in the population. Therefore, dose adjustment based on any of these parameters is not warranted. Due to the limited number of patients (*n* = 75) evaluated in the PopPK analysis, the covariate relationship will be further evaluated at the end of the phase 2 FIGHT trial. FGF2b expression was tested predose to select patients with GEA in the trial. FGFR2b status at baseline (high vs others) in patients with GEA was not a covariate for PK (Supplementary Fig. 2). In addition, there were no time-dependent changes in PK. Therefore, the PK profile should not be affected by changes in FGFR2b expression during treatment. Because no patients developed ADA after administration of bemarituzumab, there is no data to perform analysis of potential ADA impact on PK. When the impact of body weight is considered, gender does not play a significant role to impact PK parameters in general. However, the variability of CL and V_1_ for bemarituzumab cannot be fully explained by either body weight or gender and, therefore, both are significant according to the method used for the PopPK analysis and statistical criteria described in the Materials and Methods section. This result suggests that gender had impact beyond body weight although the underlying reason is not understood. This observation was previously reported for other antibodies [28, 30, 31].

The empirical target *C*_*t*rough_ of 60 µg/mL for bemarituzumab was to achieve > 95% receptor occupancy based on in vitro data, including receptor occupancy using multiple FGFR2-amplified gastric cancer cell lines. Although there is no robust clinical data to support the selection of the empirical target *C*_trough_ for bemarituzumab, clinical data indicated that at 60 µg/mL, > 90% of nonlinear clearance was projected to be saturated based on *K*_m_ obtained from the PopPK analysis. When *K*_m_ data from the clinic is available, it can be used directly to estimate target *C*_trough_. In addition, all 6 patients with GEA and confirmed partial responses in the phase 1 monotherapy trial (5 with high FGFR2b overexpression), regardless of dose level, achieved the desired target *C*_trough ss_ of ≥ 60 µg/mL although no dose-efficacy relationship was identified because of limited data [14 and Five Prime Therapeutics data on file]. It is understood that more clinical data is needed to support the empirical target *C*_trough_ and it is expected that the exposure–response relationship analysis post FIGHT trial should provide that data. For blocking antibodies, the *C*_trough_ has generally been used to select the target dose to maximize receptor occupancy throughout the treatment cycles [18–20]. Using trastuzumab as an example, two mechanisms of action are described: inhibition of the proliferation of human tumor cells that overexpress Her2 and ADCC. The minimum desired *C*_trough_ for trastuzumab in the clinic was > 10 µg/mL based on nonclinical data, which theoretically achieved ≥ 99% receptor saturation in blood based on its affinity for Her2 (0.1 nM) and < 50% receptor saturation based on its affinity for FcγRIIIa (V158) (252 nM) [19, 32, 33], respectively. In the clinic, the observed mean *C*_trough ss_ is 79 µg/mL, which is projected to achieve 99% receptor saturation of Her2, but not of FcγRIIIa [34]. Similar to trastuzumab, bemarituzumab also has two mechanisms of action: blocking FGFR2b binding to its FGF ligands and enhanced ADCC. Therefore, *C*_trough_ was used as an exposure target to saturate FGFR2b receptors during the dose interval.

The two dose cohorts in the phase 1 part of the FIGHT trial were 6 mg/kg Q2W and 15 mg/kg Q2W with a single dose of 7.5 mg/kg on Cycle 1 Day 8. As advanced stage GEA is an aggressive disease with standard chemotherapy providing a median of only 6 months of disease control with a worse prognosis expected in patients whose tumors overexpress FGFR2b, a method to shorten the time to achieve target *C*_trough_ levels was deemed clinically important. In the single agent phase 1 FPA144-001 trial, 15 mg/kg was the highest dose tested. Simulations using PopPK parameters were performed to estimate the doses required to achieve target *C*_trough_. To maximize efficacy, simulation of the dosing regimen of 15 mg/kg q2w with a single dose of 7.5 mg/kg on Cycle 1 Day 8 showed that 98% of patients could be expected to achieve the target *C*_trough_ level of 60 μg/mL on Day 15 and 96% of patients could be expected to maintain the target *C*_trough ss_. To minimize a potential safety risk which could be associated with an increase in *C*_max_, the addition of a single 7.5 mg/kg dose on Day 8 was instituted to maintain the projected *C*_max_ for this regimen within the level previously shown to be safe during the single agent study FPA144-001. It was anticipated that the addition of a single dose of 7.5 mg/kg on Cycle 1 Day 8 would shorten the time to achieve the target *C*_trough_ which was thought to be clinically important in the early control of an aggressive tumor such as GEA. Indeed, target *C*_trough_ was achieved by all patients in the phase 1 portion of the FIGHT trial on Day 15 using this dose and regimen. This PopPK analysis and clinical findings supported the selection of dose and schedule for initiation of the phase 2 randomized, double-blind portion of the global FIGHT trial which is currently ongoing worldwide [24].

## Electronic supplementary material

Below is the link to the electronic supplementary material.

Supplementary file1 (DOCX 346 kb)
