# Supplementary material for: Population pharmacokinetic analysis of phase 1 bemarituzumab data to support phase 2 gastroesophageal adenocarcinoma FIGHT trial
Source: Cancer Chemother Pharmacol. 2020 Sep 23;86(5):595–606. doi: 10.1007/s00280-020-04139-4 (PMC7561547; doi:10.1007/s00280-020-04139-4)

# Supplementary Figures

Fig. 1 Basic goodness-of-ﬁt plots for the ﬁnal bemarituzumab pharmacokinetic model. Observed bemarituzumab concentrations (µg/mL) are plotted versus population and individual predictions (top). Conditional weighted residuals (CWRES) are plotted against time and population predictions (bottom). The black open circles represent the data points. The solid red line in each plot is the line of identity and the blue dashed lines are |CWRES| = 4.

Fig. 2 The impact on Vmax by FGFR2b expression in patients with gastric and gastroesophageal junction adenocarcinoma. Box blots were used to compare individual random effects (ETAs) for Vmax in FGFR2b high patients (10% of tumor cells with 3+ membranous staining) with GEA versus all other patients with GEA. The top and bottom ends of the box plot represent the 25th and 75th percentile (the lower and upper quartiles, respectively). The median is represented by the horizontal white line in the middle of each blue box. The bars extending from the ends of the box to the outermost data represent 1.5 × the upper or lower interquartile range, respectively.

Supplementary Fig. 1


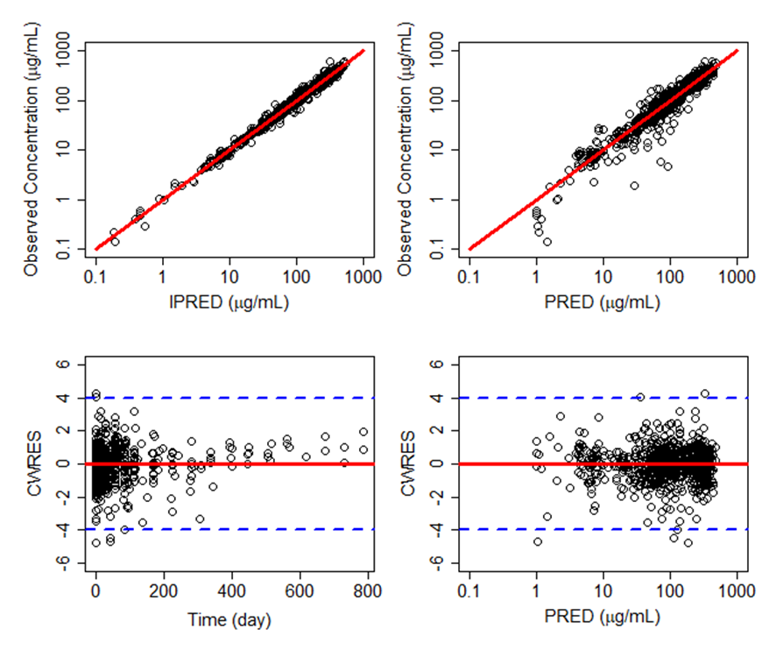


Supplementary Fig. 2


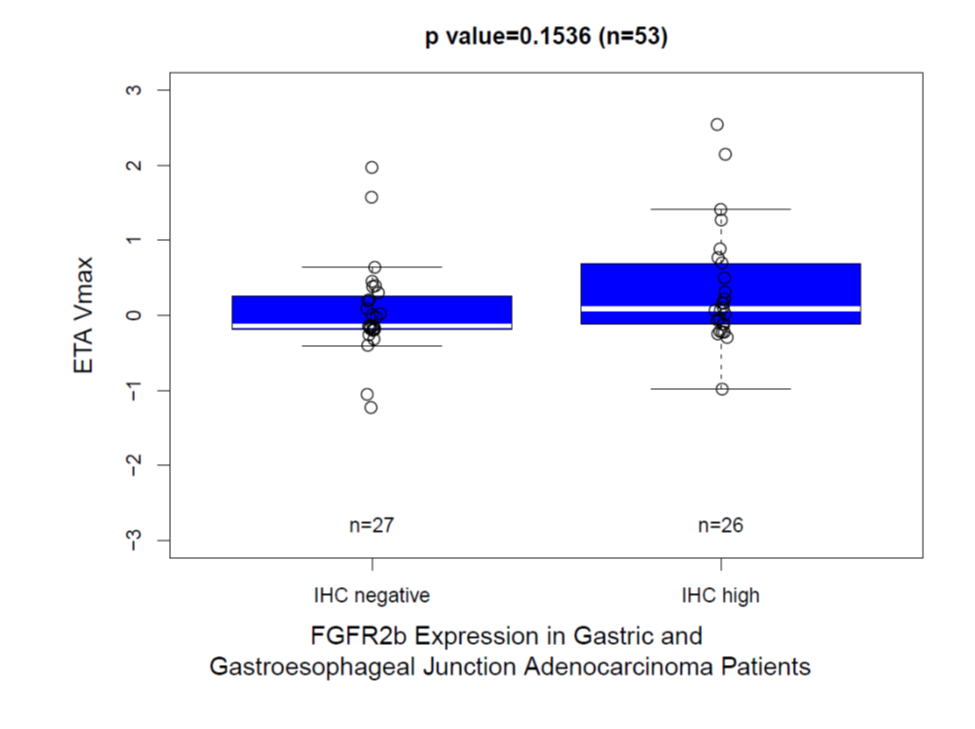

Supplement: Supplementary file 1 — Supplementary file1 (DOCX 346 kb) [file 280_2020_4139_MOESM1_ESM.docx]
